# Modeling Visual Exploration in Rhesus Macaques with Bottom-Up Salience and Oculomotor Statistics

**DOI:** 10.3389/fnint.2016.00023

**Published:** 2016-06-30

**Authors:** Seth D. König, Elizabeth A. Buffalo

**Affiliations:** ^1^Wallace H. Coulter Department of Biomedical Engineering at the Georgia Institute of Technology and Emory UniversityAtlanta, GA, USA; ^2^Yerkes National Primate Research CenterAtlanta, GA, USA; ^3^Graduate Program in Neuroscience, University of WashingtonSeattle, WA, USA; ^4^Washington National Primate Research CenterSeattle, WA, USA; ^5^Department of Neurology, Emory University School of MedicineAtlanta, GA, USA; ^6^Department of Physiology and Biophysics, University of WashingtonSeattle, WA, USA

**Keywords:** salience, bottom-up, attention, free viewing, random walk, natural behavior

## Abstract

There is a growing interest in studying biological systems in natural settings, in which experimental stimuli are less artificial and behavior is less controlled. In primate vision research, free viewing of complex images has elucidated novel neural responses, and free viewing in humans has helped discover attentional and behavioral impairments in patients with neurological disorders. In order to fully interpret data collected from free viewing of complex scenes, it is critical to better understand what aspects of the stimuli guide viewing behavior. To this end, we have developed a novel viewing behavior model called a Biased Correlated Random Walk (BCRW) to describe free viewing behavior during the exploration of complex scenes in monkeys. The BCRW can predict fixation locations better than bottom-up salience. Additionally, we show that the BCRW can be used to test hypotheses regarding specific attentional mechanisms. For example, we used the BCRW to examine the source of the central bias in fixation locations. Our analyses suggest that the central bias may be caused by a natural tendency to reorient the eyes toward the center of the stimulus, rather than a photographer's bias to center salient items in a scene. Taken together these data suggest that the BCRW can be used to further our understanding of viewing behavior and attention, and could be useful in optimizing stimulus and task design.

## Introduction

Recently, there has been a growing interest in studying biological systems in natural settings, in which experimental stimuli are less artificial and behavior is less controlled (Felsen and Dan, 2005; Hayhoe and Ballard, 2005; Meister and Buffalo, 2015). Behavioral paradigms using free viewing in primates have uncovered novel signals in the hippocampal formation related to recognition memory, spatial representations, visual exploration, and saccadic eye movements (Killian et al., 2012; Hoffman et al., 2013; Jutras et al., 2013). Additionally, several recent studies in humans have illustrated the utility of complex scenes and movies in studying changes in attention and behavior in patients with neurological disorders (Smith et al., 2006; Crutcher et al., 2009; Mannan et al., 2009; Tseng et al., 2013; Zola et al., 2013; Wang et al., 2015). In order to fully interpret these data, it is critical to better understand what aspects of the stimuli guide viewing behavior. To this end, we have developed a novel foraging model to describe free viewing behavior during the exploration of complex scenes in monkeys. This model allows us to predict where the monkeys will fixate.

A variety of viewing behavior models exist which can be broadly classified as “top-down” or “bottom-up” (see Kimura et al., 2013; Bylinskii et al., 2015 for recent reviews on human models of attention). Top-down models predominately focus on search-based tasks in which participants attempt to find a target among distractors in a complex environment (Wolfe, 1994). Conversely, bottom-up models predominately utilize salience in pop-out search tasks and in free viewing of complex scenes (Itti et al., 1998; Parkhurst et al., 2002; Bruce and Tsotsos, 2005; Elazary and Itti, 2008; Judd et al., 2011; Wilming et al., 2011; Zhao and Koch, 2011). The success of individual models appears to depend on various experimental factors including task demands and the types of stimuli used (Turano et al., 2003; Henderson et al., 2007; Shic and Scassellati, 2007). It is becoming increasingly popular to incorporate aspects of both bottom-up and top-down mechanisms to create hybrid models that can predict behavior better than either mechanism separately (Lee et al., 2005; Walther and Koch, 2006; Zhang et al., 2008; Kollmorgen et al., 2010; Nordfang et al., 2013). While the majority of these models were designed to predict human behavior, several studies have shown that these models sufficiently predict behavior in non-human primates as well (Einhauser et al., 2006; Berg et al., 2009).

Most attention models are deterministic and often employ a winner-take-all algorithm to interpret attention maps. However, viewing behavior is inherently stochastic and can vary within and across observers. Several stochastic models of viewing behavior have been proposed, including a few which model realistic eye movements (Verghese, 2001; Boccignone and Ferraro, 2004, 2014; Harel et al., 2006; Rutishauser and Koch, 2007; Barthelmé et al., 2013; Zehetleitner et al., 2013). While these models address the variability found in natural behavior, it is difficult to directly apply some of these models to free viewing of complex scenes. Further, some of these models do not include realistic models of eye movement statistics making it difficult to test hypotheses regarding changes in attention and viewing behavior.

To address these limitations, we propose a novel model of viewing behavior for complex scenes called a Biased Correlated Random Walk (BCRW). We build the BCRW model under the hypothesis that the constraints of the oculomotor system interact with the arrangement of the salient regions of the image to guide behavior. To this end we use a simple random walk process to construct a foraging model of viewing behavior in which observers forage for salience as a simple surrogate of visual information. The BCRW is essentially a model of eye movements and provides a method for interpreting salience maps or other forms of attention maps.

To demonstrate the utility of the BCRW, we show that the BCRW can help adjudicate between competing hypotheses regarding the central bias in fixation locations commonly observed during the viewing of complex scenes. A large number of studies have observed a central bias in fixation locations with humans typically producing a stronger central bias than monkeys (Parkhurst et al., 2002; Berg et al., 2009; Wilming et al., 2011; Wang et al., 2015). The central bias has been hypothesized to be driven by a variety of factors including a photographer's bias (the tendency of photographers to center objects of interest in a picture), the use of a central fixation target to initiate trials, the centering of stimuli relative to subjects, a natural tendency for subjects to re-center the eyes, and the fact that subjects typically make small amplitude saccades resulting in the location of gaze remaining near the center of the image (Tatler, 2007; Tseng et al., 2009; Bindemann, 2010).

Here, we hypothesized that the central bias in fixation locations is caused by the interaction between the arrangement of salient regions in complex scenes (i.e., photographer's bias) and the statistics of the oculomotor system. We offer empirical and modeling evidence using the BCRW which suggest that the photographer's bias and statistics of the oculomotor system are not sufficient to explain the central bias.

## Methods

### Behavioral task and eye tracking

**Figure 1:**
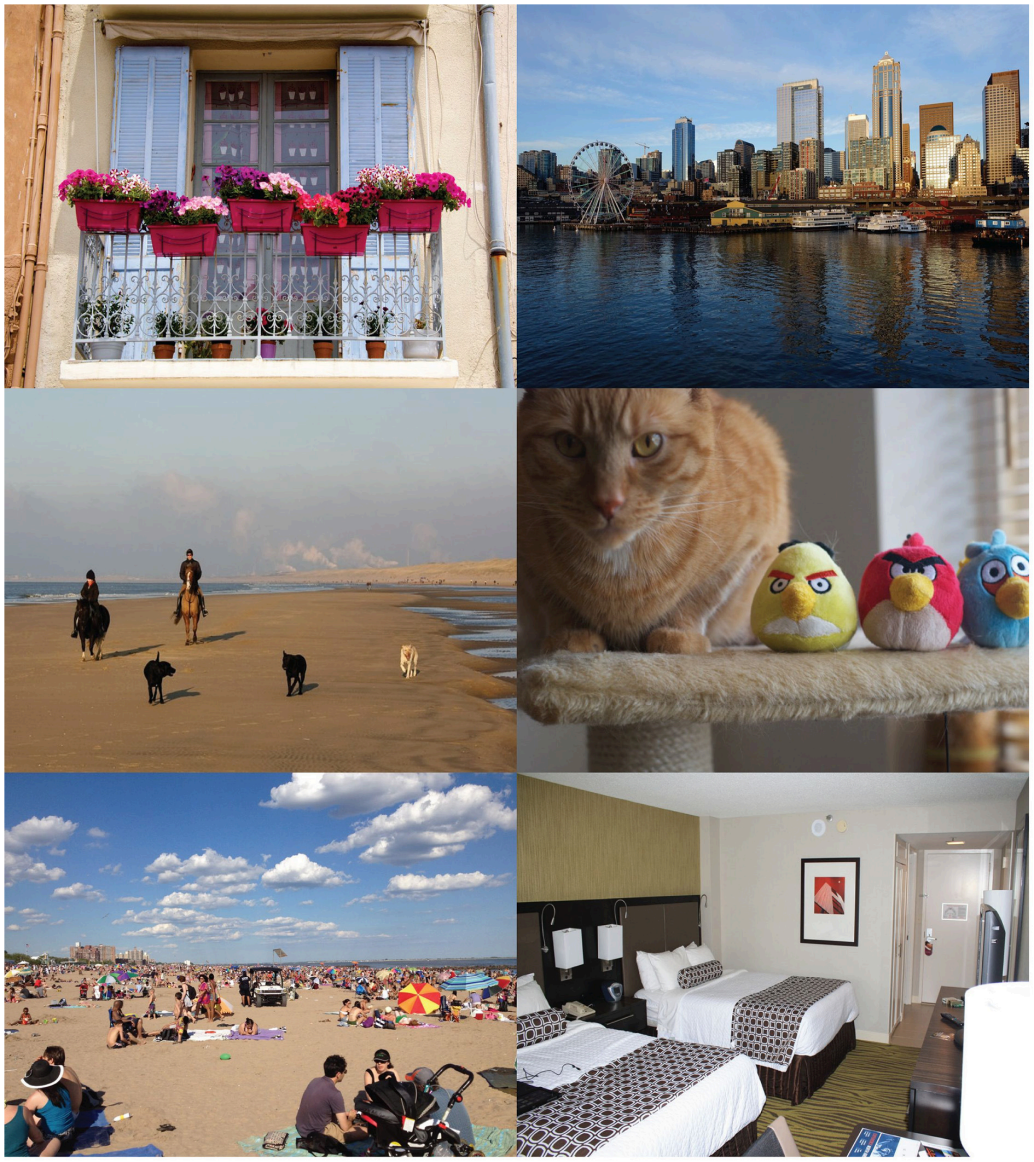
**Example images: These images range in complexity and may include images of humans, non-human primates, animals, urban scenes, outdoor scenes, or indoor scenes from a variety of vantage points**. All images were taken from Flickr. Images were captured by (from top to bottom and left to right): Amanda, Sean Munson, Marcel Oosterwijk, Melinda Seckington, Luna Park NYC, Richard Franklin. These images are reproduced under creative commons licenses.

Scan paths were obtained at 200 Hz using an infrared eye tracker (ISCAN) from four male rhesus macaques who freely viewed complex images. Monkeys were head-fixed in a dimly illuminated room and positioned 60 cm away from a 19 inch CRT monitor with a refresh rate of 120 Hz. Monkeys were presented a total of eight image sets with each image set containing 36 novel images. Images were 600 by 800 pixels large and subtended 25° by 33° of visual angle (dva). Images were taken from Flickr. These images ranged in complexity and included animals, humans, architecture, outdoor scenes, indoor scenes, and manmade objects (Figure 1).

Each trial began with the presentation of a 0.5 dva cross in the center of the screen. The monkey was required to fixate the cross, within a fixation window with a width of 4 dva, for 1 s. Immediately following a successful fixation, an image was displayed and the monkey was allowed to freely view the image for 10 s of cumulative looking time. We analyzed the first 10 s of viewing behavior, regardless of the length of the image presentation. Because viewing behavior data were collected for a separate experiment, a second image presentation followed the novel image presentation. Data for these trials were not analyzed as part of the current study; only novel image trials were used for analysis. Pairs of image presentations were interleaved with calibration trials.

Initial calibration of the infrared eye tracking system consisted of a nine-point manual calibration task (Jutras et al., 2013). *Post-hoc* calibration was achieved by presenting additional calibration trials between image viewing trials. Monkeys were rewarded for successful calibration trials, but were not rewarded during the image viewing periods. We excluded from further analysis any eye tracking data more than 25 pixels (1 dva) outside of the image. To account for small calibration errors at the edge of images, any fixations occurring within 25 pixels of the image were moved to the closest point on the image.

A *k*-means cluster analysis based algorithm, called Cluster Fix, detected fixations and saccades from scan paths in state space (König and Buffalo, 2014). Briefly, Cluster Fix determined the distance, velocity, acceleration, and angular velocity of the scan path. Cluster Fix found natural divisions in these four parameters using *k*-means clustering to separate time points into fixations and saccades and required minimum fixation and saccade durations of 25 and 10 ms, respectively.

All experiments were carried out in accordance with the National Institutes of Health guidelines and were approved by the Emory University Institutional Animal Care and Use Committee and by the University of Washington Institutional Animal Care and Use Committee.

### Viewing behavior statistics and time warping

**Figure 2:**
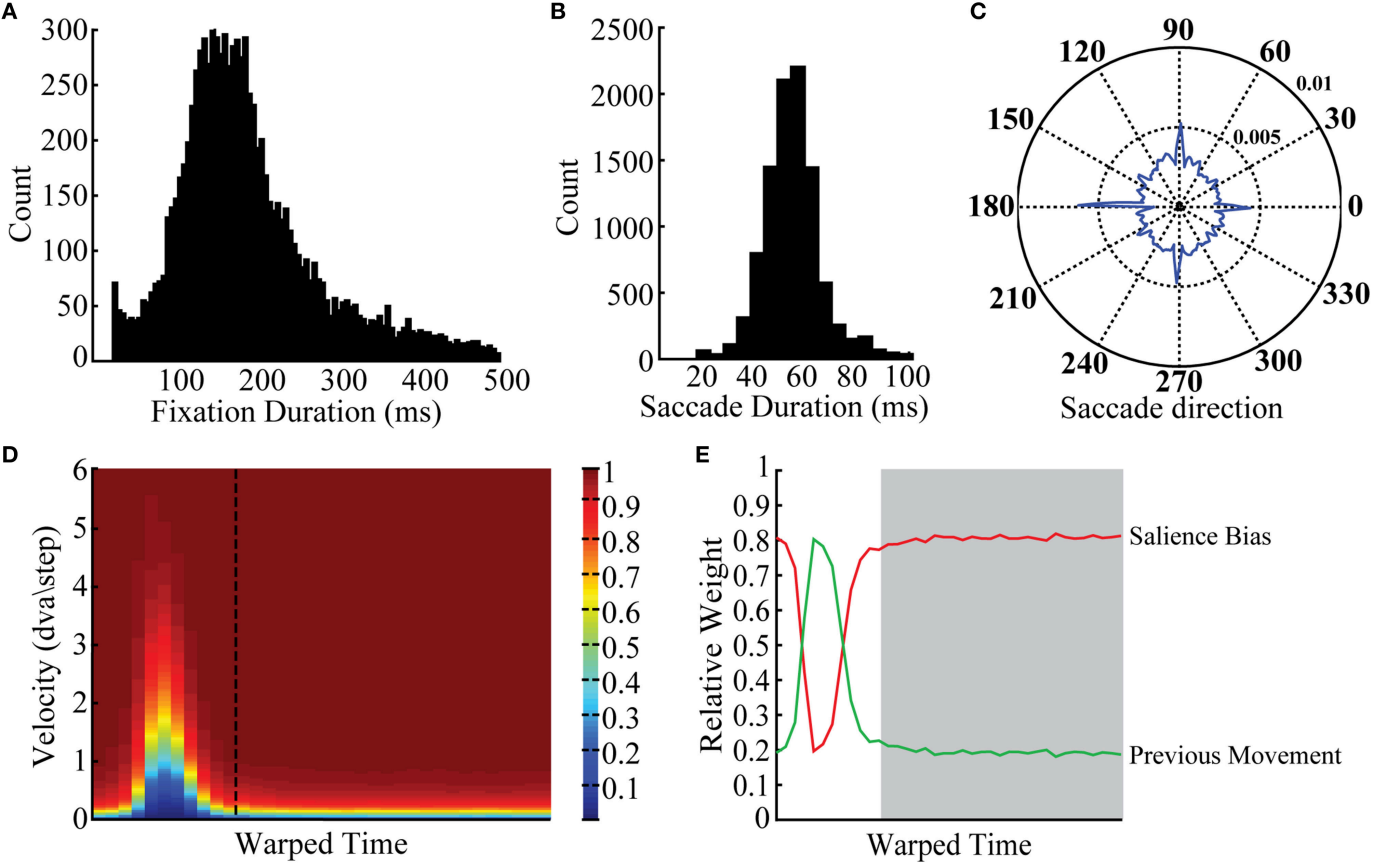
**Observed behavioral statistics**. Viewing behavior statistics incorporated into the BCRW included **(A)** fixation durations, **(B)** saccade durations, **(C)** the saccade angle leaving a fixation, **(D)** the eye movement velocity over time (dashed line is transition time from saccade to fixation), and **(E)** the relative weight of the salience bias and direction of previous movement (white background: saccade period, gray background: fixation period). The color axis for **(D)** is cumulative probability. Examples shown here are from monkey MP, but these statistics were similar across monkeys. Histograms are plotted over a limited range to illustrate the distribution; raw data were incorporated directly into the BCRW.

The eye tracking data contained ~40,000 fixations and saccades. We simulated the BCRW separately for each monkey using individual parameters for fixation durations, saccade durations, angles of saccades leaving fixations, the eye velocity over time, and relative weight of the salience bias (Figure 2). The average fixation duration and saccade duration across all monkeys was 215 and 56 ms, respectively; fixations occupied 79% of the scan path. Several eye movement statistics varied systematically by ordinal fixation or saccade number, but these phenomena were not incorporated into the BCRW.

Viewing behavior statistics were calculated directly from the raw scan paths. Cluster Fix detected the start and end of fixations and saccades. To combine fixations and saccades of different durations, we used a process called time warping (Sober et al., 2008). In particular fixation durations were not normally distributed and varied over a large range. To calculate parameter values such as persistence it was important to determine these values across all fixations and saccades. Therefore, we warped each fixation or saccade using linear spaced sampling to rescale all fixations and saccades to their respective medians. During the simulation, parameter values such as persistence were warped to fit fixation durations and saccade durations selected by the BCRW. Viewing behavior statistics generated by the BCRW are shown in Supplementary Figure 1.

### Calculating salience maps and image intensity

Salience is a bottom-up property of visual stimuli and is defined as stimulus features distinct from the background that immediately attract attention. Mathematically, we computed salience by summing color, orientation, and intensity contrast over multiple spatial scales (Itti et al., 1998). We then normalized the salience map from 0 to 1 within each image. Image intensity was also normalized from 0 to 1 within each image and was defined as the gray scale value at each pixel. Salience and image intensity chance levels were calculated as the 95% confidence intervals of randomly selected locations. The selection of the Itti, Koch, and Niebur salience model was based on the simplicity of the model as well as the inclusion of biologically inspired contrast filters.

### Biased correlated random walk (BCRW)

**Figure 3:**
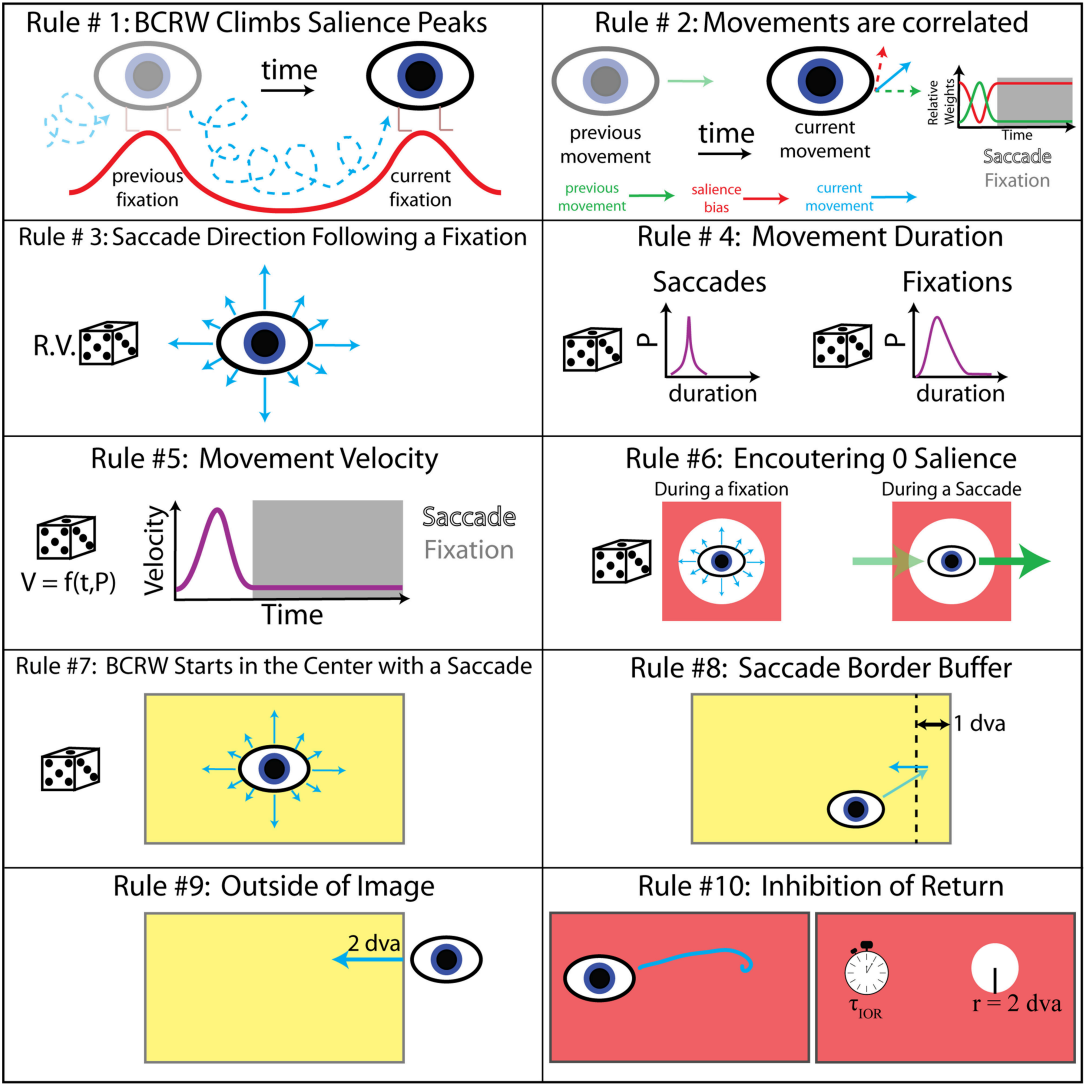
**BCRW rules**. Ten rules dictated the velocity, direction, and duration of movement. The values for rules 8–10 were determined by a parameter sweep. *R.V*., random variable; *P*, probability; *f*, function; *t*, time; *V*, velocity; τ_IOR_, time constant of IOR; and *r*, radius.

The BCRW consisted of a salience map and a random walk process informed by viewing behavior statistics. See Figure 3 for a summary of rules that dictated movement in the BCRW, and see Table 1 for pseudo code. In the BCRW, a bias term and the direction of previous movement competed to influence the walk (Crone and Schultz, 2008). The bias term caused the walk to move toward the most salient regions of the image. The direction of previous movement was used to emulate saccadic eye movements in which the eyes move in the same direction until the eyes approach a fixation target. A weighted average of the previous movement angle and the gradient of the salience map determined the direction of current movement. We estimated the weighting term, which we call persistence, from the probability that the scan paths did not change direction by more than 45°; persistence was greater during a saccade than during a fixation.

**Table 1:** **BCRW pseudo-code**.

1. Smooth salience map with Gaussian filter 2. Calculate gradient of salience map to get direction of salience bias 3. Start eye at the center of the image 4. **Do** Simulate Saccade() Simulate Fixation() **While** simulated time < 10 s Simulate Saccade() a. Randomly select a saccade duration from the observed distribution b. Warp observed eye velocity distribution and persistence to selected saccade duration c. **For** each time step during the saccade **If** this is the first time step in the saccade Randomly select a velocity from the eye velocity distribution Randomly select a saccade angle leaving a fixation from observed distribution Move selected velocity and direction **Else** Randomly select a velocity from the eye velocity distribution If salience at current location is 0 Current direction = previous movement direction **Else** Direction of current movement = persistence*previous movement direction + (1–persistence)*direction of salience bias Move selected velocity and calculated direction **If** saccade approaches within 1 dva of the border of the image Move in direction away from the border of the image **Else If** the saccade leaves the image Move 2 dva back into the image Simulate Fixation() Randomly select a fixation duration from the observed distribution Warp observed eye velocity distribution and persistence to selected fixation duration **For** each time step during the fixation **If** salience at current location is 0 Set salience bias to random direction Randomly select a velocity from the eye velocity distribution Direction of current movement = persistence*previous movement direction + (1–persistence)*direction of salience bias Move selected velocity and calculated direction **If** this is the last time step in the fixation Estimate fixation location as average position over the last 5 time steps (25 ms) Set salience at fixation location and surrounding area (IOR_radius_ = 2 dva) to 0 Recover salience at prior fixation locations if locations were fixated 17 fixations ago (i.e., 1/τ_IOR_)

The direction of movement dictated by the salience map always pointed in the direction of the highest salience. In other words the BCRW climbed salience peaks. To assist the BCRW in climbing salience peaks, the salience maps were smoothed by a Gaussian filter with a standard deviation of 1/2 dva. In the case where the walk encountered 0 salience, walks producing saccades continued in the previous direction while walks producing fixations moved in a random direction dictated by the probability distribution function (PDF) of saccade angles leaving a fixation.

All distributions of durations, directions, and velocity of movements in the BCRW were determined directly from the observed scan paths for each monkey individually. A PDF of saccade angles leaving a fixation dictated the saccade direction at the start of the BCRW and following a fixation. Parallel to the monkey experiments, the BCRW started at the center of the image. PDFs of fixation and saccade durations as well as PDFs of fixation and saccade velocity, which were functions of time and velocity, determined the duration and velocity of movement in the BCRW. The persistence term and PDFs of fixation and saccade velocity were time warped to the median length of all fixations and saccades, respectively. Since the duration of fixations and saccades changed randomly within the BCRW these movement distributions were then warped to the length of the fixation or saccade determined during the BCRW.

The BCRW produced a saccade followed by a fixation for a predetermined duration based on PDFs of saccade and fixation durations, respectively. At the end of the fixation period, the fixation location was determined to be the mean location of the simulated scan path for the last 25 ms of the fixation. Averaging over the last 25 ms accounted for any positional jitter as well as the fact that the BCRW could systematically drift toward local salient peaks (Supplementary Figure 2). The salience surrounding the fixation location within the area of inhibition of return (IOR) was then set to 0 (see below). The BCRW then “reset” and produced the next fixation and saccade pair until the scan path had been simulated for a total of 10 s. The BCRW had a temporal resolution of 5 ms similar to the acquisition rate of the eye tracker (200 Hz). For each monkey and each image, we simulated the BCRW 100 times using that monkey's behavioral statistics to obtain a prediction of fixation locations.

The BCRW contained four additional parameters not derived from viewing behavior statistics: a border buffer rule, border saccade distance rule, the time constant of IOR, and the area effected by IOR. A parameter sweep estimated these parameters (Table 2). These parameters were fit across all monkeys and did not vary individually. For the parameter sweep, we used Kullback–Leibler divergence (KL divergence) to compare observed fixations to predicted fixation locations from 10 simulations of the BCRW for each image and monkey. The border buffer rule stated that whenever a saccade approaches within 1 dva of the image border then the saccade must move in the direction opposite to the border. The border saccade distance rule stated that whenever the BCRW left the image and crossed the image border, the BCRW must move in the direction opposite to the border with a distance that is at least 2 dva. The radius for the area of IOR was found to be 2 dva and the optimal time constant for IOR was found to be 1/17th. The time constant of IOR required a certain number of fixations, the reciprocal of this value, to occur before the salience returned to its original value at a previous fixation location. IOR models the consumption and recovery of visual information at fixation locations.

**Table 2:** **Parameter Sweep values**.

**Parameter name**	**Tested values**	**Value of best fit**
Border buffer (dva)	0.04, 0.4, 1, 2, 4	1 dva
Border saccade distance (dva)	0.4, 1, 2, 4, 8	2 dva
Time constant of IOR (1/# of fixations)	0, 1/50, 1/35, 1/25, 1/17, 1/12, 1/7, 1/3, 1	1/17
Area of IOR (radius in dva)	0, 1, 2, 4	2 dva

### Correlated random walk (CRW)

To determine how important the salience bias was for predicting viewing behavior, we created a correlated random walk (CRW) model without a salience bias. Because we use IOR to model the consumption of information with salience being a proxy for information, the CRW does not contain IOR. In the CRW persistence determined the relative weight of the direction of the previous movement and a random direction.

### KL divergence and ROC analysis

KL divergence was used to compare the PDFs of observed fixation locations to the PDFs of the predicted fixation locations. The observed and predicted fixation PDFs were calculated by combining the fixation locations for all four monkeys who viewed the same image into a single fixation matrix of the same size as the image. The fixation matrix was marked with a one where fixations occurred and then smoothed by a 2D Gaussian filter with a standard deviation of 1 dva. The smoothing accounted for small errors in the eye tracking system and natural variability in fixation location (Wilming et al., 2011); the standard deviation of the eye tracking error was typically < 1/4 dva. Binning the fixation matrix into 1 × 1 dva bins created a new matrix containing 24 × 32 bins. In the case where a bin contained no fixations, the bin's value was replaced with the lowest value defined in MATLAB, eps (2^−52^). Finally, the fixation PDF was estimated by dividing the binned fixation matrix by the sum of the entire matrix. The predicted fixation PDF for the salience map and image intensity map were derived directly from these maps by binning the maps and then dividing the maps by their resulting sum.

KL divergence was calculated from the binned PDFs using the following equation:

(1) $$\begin{array}{rc} D_{KL}=D_{KL}\left(P||Q\right)+D_{KL}\left(Q||P\right)=\underset{i,j}{\sum }\ln\;\left(\frac{P\left(i,j\right)}{Q\left(i,j\right)}\right)P\left(i,j\right)\\ + & \underset{i,j}{\sum }\ln\;\left(\frac{Q\left(i,j\right)}{P\left(i,j\right)}\right)Q\left(i,j\right) \end{array}$$

where *D*_*KL*_ is the symmetric form of KL divergence, *P* is the first fixation PDF, *Q* is the second fixation PDF, *i* is the bin row, and *j* is the bin column.

A Receiver Operating Characteristic (ROC) analysis allowed us to determine whether the models could discriminate between fixated and non-fixated locations. We used ROC analysis to compare the distribution of BCRW, salience map, or image intensity map values at fixated locations to the distribution of values at random locations. We then calculated the AUROC (Area Under the Receiver Operating Characteristic Curve) from the ROC curves. AUROC values close to 1.0 suggest that these models are good at discriminating between fixated and non-fixated locations while an AUROC value of 0.5 suggests these models discriminate between fixated and non-fixated locations at chance levels.

There exist a variety of methods for determining model fitness and in particular for visual salience models (Wilming et al., 2011). Compared to a ROC analysis, KL divergence better identifies excellent models predicting the probability of fixation in certain locations because KL divergence weighs higher fixation probabilities heavier than low fixation probabilities. This notion is important because certain areas (theoretically the most salient regions) are fixated repeatedly as compared to other areas which are less likely to be fixated even once. In contrast to KL divergence, a ROC analysis weighs each location equally regardless of the probability of fixation. Thus ROC analysis is better at assessing a model's ability to predict fixation locations regardless of the fixation probability. Another benefit of a ROC analysis is that ROC analysis does not require corrections for locations with zero fixation probability and thus smaller sample size affects ROC measures less. Because both methods have their advantages and disadvantages, we used both to test each model's goodness of fit.

### Shift task

To test the central bias hypothesis, we used data from two monkeys performing free viewing of complex images as part of a separate experiment. Data for this experiment were obtained separately from one monkey used in the previous experiment and an additional fifth monkey. In this task, the image position was shifted left or right of the center of the monitor by 2 dva. Importantly, the initial fixation cross was presented at nine different points, located along the border of the image in the eight cardinal and intermediate directions and one at the center of the image. Compared to the central fixation cross condition, the fixation cross was shifted vertically up to ~7 dva and horizontally up to ~15 dva.

For the examination of the central bias hypothesis, only the viewing behavior statistics from this task were incorporated into the BCRW. We simulated the BCRW 100 times for a total of 360 images for each monkey. Three or four image sets were used for each monkey with image sets containing 120 or 90 novel images, respectively. Images were 378 by 756 pixels large and subtended 16 by 32 dva.

## Results

### Fitness of salience and BCRW models

**Figure 4:**
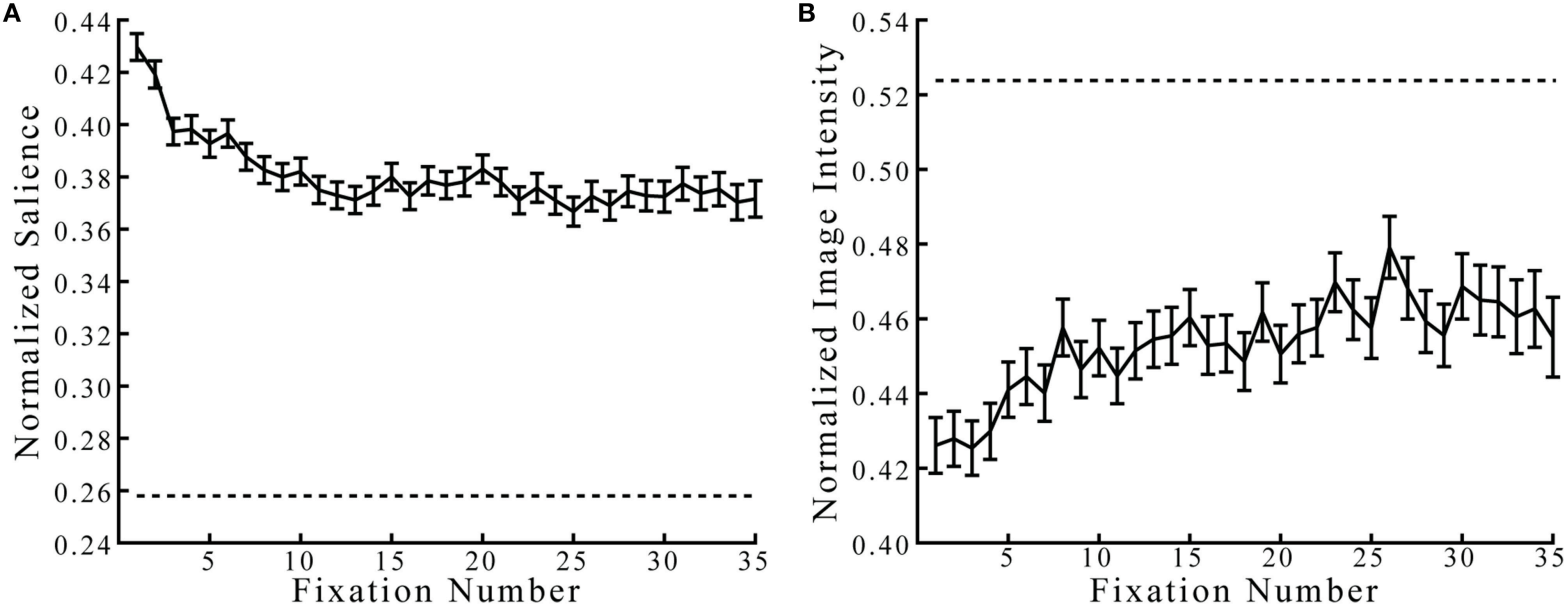
**Salience and image intensity at observed fixation locations**. **(A)** Observed fixations occurred at locations with salience values higher than would be expected by chance (dashed lines represent chance levels). **(B)** Fixations occurred at locations with image intensity values lower than expected by chance. Error bars represent mean ± *SEM*.

Because the BCRW relies heavily on salience, we first determined whether observed fixation locations occurred at more salient locations than expected by chance (Figure 4). Not surprisingly, fixations occurred at locations with salience values higher than expected by chance, while fixations occurred at locations with image intensity values lower than expected by chance (*z*-test, both *p'*s < 0.001). The mean salience at all fixation locations was 0.3806, and the mean image intensity was 0.4533. The salience and image intensity chance levels were 0.2579 and 0.5238, respectively. Salience at fixation locations was higher during the first few fixations compared to later on in the viewing period, and salience at later fixations appeared to approach an asymptote. Similarly, fixations occurred at brighter locations later in the viewing period compared to earlier in the viewing period.

**Figure 5:**
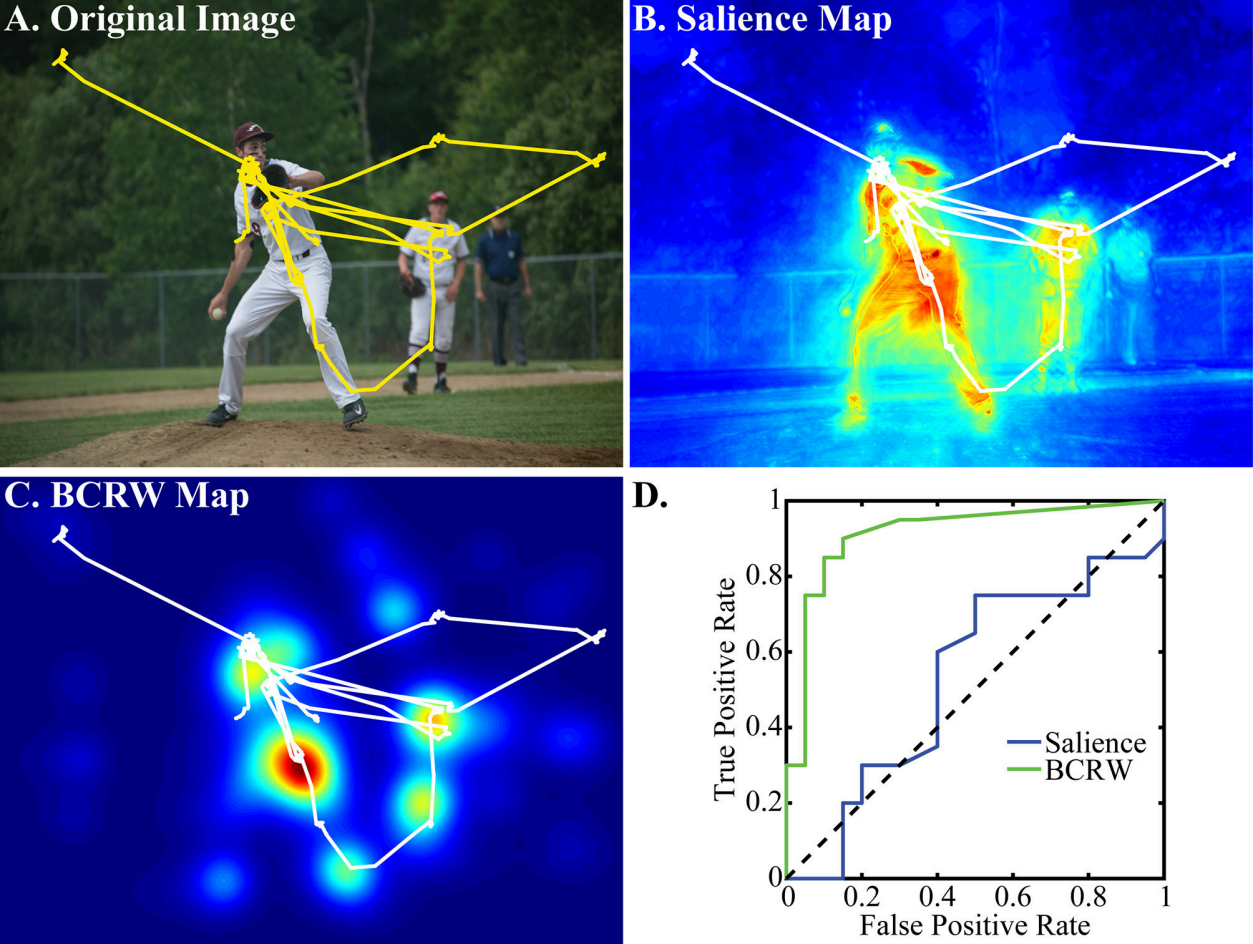
**Examples of observed scan path, salience map, and BCRW map**. **(A)** The observed scan path (yellow) overlaying the viewed image shows that the monkey attends to many of the objects in the scene. **(B)** The observed scan path (white) overlaying the salience map shows that fixations occurred in many, but not all of the salient regions of the image. **(C)** The BCRW performed well at predicting fixation locations. **(D)** A ROC analysis demonstrated that the BCRW was better than salience at discriminating between fixated and non-fixated locations for this image. This image was captured by Foxcroft Academy and is reproduced under creative commons licenses.

Individual scan paths revealed that fixations occurred in many but not all of the salient regions of the image (Figure 5). Accordingly, we built the BCRW to simulate this variability in scan paths. We used KL Divergence and ROC analysis to compare the ability of the BCRW, salience, and image intensity maps to predict fixation locations.

**Figure 6:**
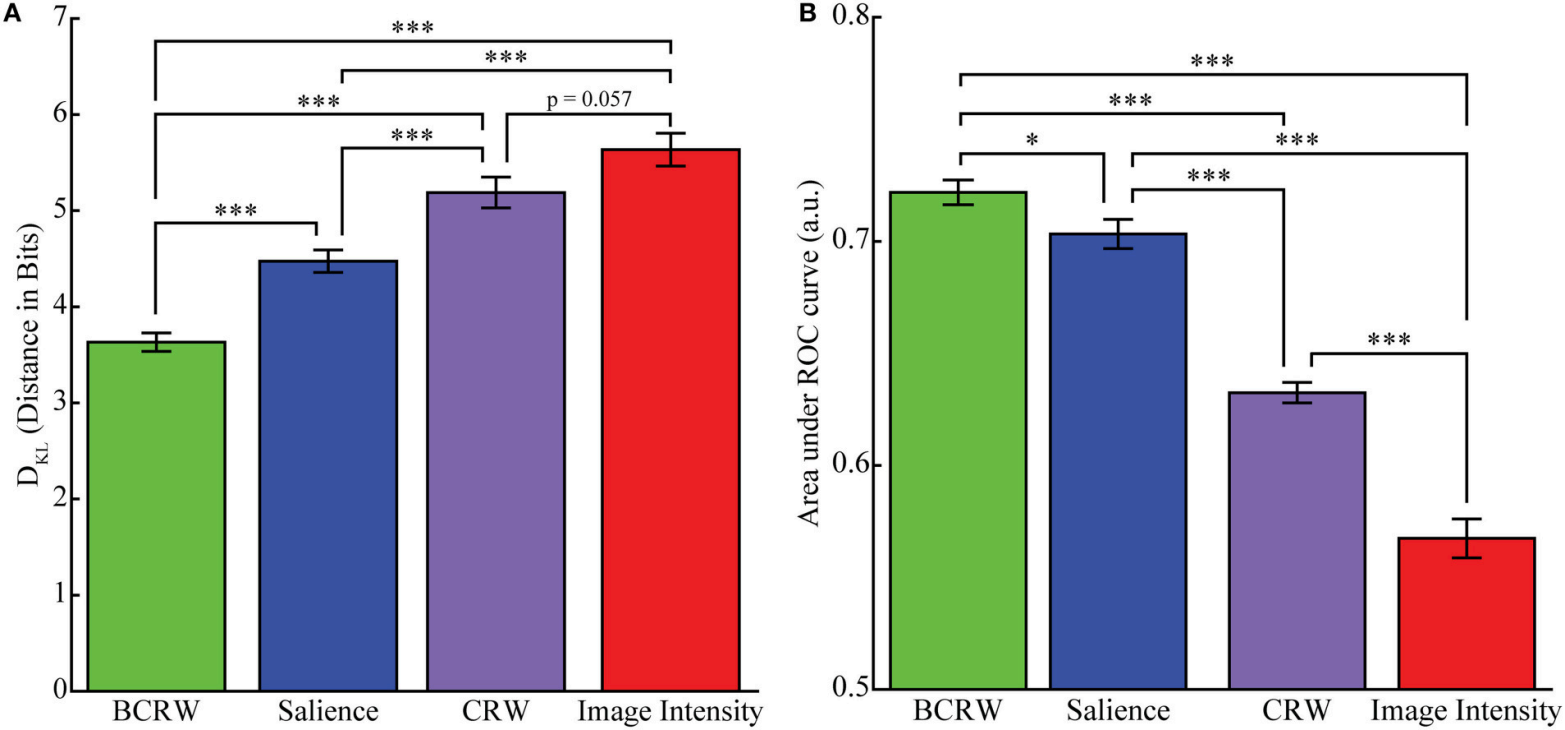
**Comparing models for predicting fixation locations. (A)** KL Divergence analysis showed that the BCRW predicted fixation locations better than salience and image intensity, and salience predicted fixation locations better than image intensity. The CRW was worse at predicting fixation locations than the BCRW and salience but not image intensity. **(B)** The mean AUROC was significantly higher for the BCRW than for salience or image intensity, and the AUROC for salience was higher than for image intensity. The CRW was worse at predicting fixations than the BCRW and salience but better than image intensity. The CRW, BCRW, salience, and image intensity can all be useful for discriminating between fixated and non-fixated locations because the AUROC for all maps were significantly greater than chance. Error bars represent mean ± *SEM*. (^*^p < 0.05 and ^***^p < 0.001).

The KL divergence analysis showed that the distance between the fixation PDF predicted by the BCRW and observed fixation PDF was significantly shorter than the distance between the fixation PDF predicted by the salience map and the observed fixation PDF (*t*-test, *p* < 0.001), and was significantly shorter than the distance between the fixation PDF predicted by the image intensity map and the observed fixation PDF (*t*-test, *p* < 0.001; Figure 6). Additionally, the fixation PDF predicted by the salience map was significantly closer to the observed fixation PDF than the fixation PDF predicted by the image intensity map (*t*-test, *p* < 0.001). The mean distance between observed fixations and fixations predicted by the BCRW, salience, and image intensity maps was 3.63, 4.47, and 5.63 bits, respectively. These results indicated that the BCRW performed better than salience or image intensity maps.

Similar results were obtained from a ROC analysis: the BCRW discriminated between fixated and random locations better than salience (*ks*-test, *p* = 0.02) and better than image intensity (*ks*-test, *p* < 0.001). Additionally, salience discriminated between fixated and random locations better than image intensity (*ks*-test, *p* < 0.001; Figure 6). The mean AUROC for the BCRW, salience, and image intensity was 0.722 0.704, and 0.567, respectively. All AUROCs were significantly greater than chance (*z*-test, all *p'*s < 0.001).

To determine whether the salience bias was important for predicting fixation locations we generated scan paths using a CRW. The CRW predicted uniform distribution of fixation locations except near the edge of the image and does not represent the observed fixation data (Supplementary Figure 2). KL divergence analysis showed that the CRW (mean of 5.19 bits) was significantly worse at predicting fixation locations than the BCRW or salience (*t*-test, *p* < 0.001), but the CRW was marginally better than image intensity (*t*-test, *p* = 0.057). A ROC analysis showed similar results. The AUROC for the CRW (mean of 0.632) was significantly worse than the BCRW and salience (*ks*-test, *p* < 0.001) but significantly better than image intensity (*ks*-test, *p* < 0.001).

### How well can a central bias and interobserver consistency predict fixations?

Interobserver consistency in humans is generally very high and is often considered the upper limit on model performance. To calculate interobserver consistency, we compared fixation locations of an individual monkey to the remaining group of monkeys using a ROC analysis. We did this for every combination of individuals and groups. The average AUROC was 0.748 which was significantly better than chance (*z*-test, *p* < 0.001). Interestingly, AUROC values ranged from 0.467 to 0.929 suggesting that some images were viewed consistently while others were viewed dissimilarly. Interobserver consistency was significantly better at predicting fixation locations than image intensity, salience, and the BCRW (*t*-test, all *p'*s < 0.001) although this difference was modest for the BCRW (approximately a 4% change).

**Figure 7:**
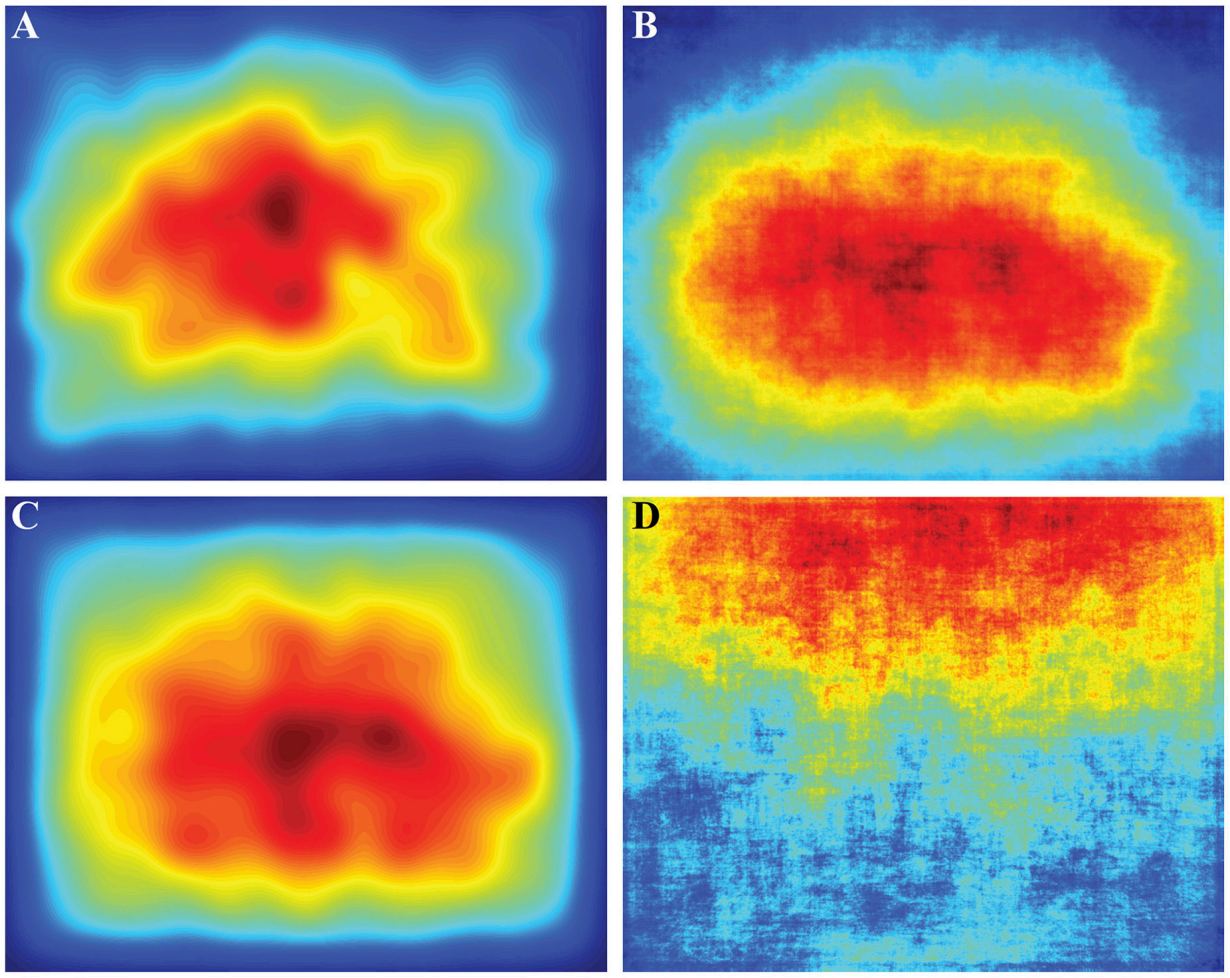
**Average probability density functions (PDFs) across all images. (A)** The average observed fixation PDF contained an evident central bias. The initial fixation to start the trial was not included in this PDF. **(B)** The salience map contained a central bias that appeared similar to the observed fixation PDF. **(C)** The average BCRW map also contained a strong bias toward the center. **(D)** The average image intensity map did not contain a central bias and did not represent the fixation PDF well. Averaging across all images revealed that the top of the image was brighter than the bottom of the image. Many images contained the horizon in the upper portion of the images which contributed to this phenomenon.

To calculate the influence of the central bias on fixation location we compared observed fixation locations to the average observed fixation PDF, salience PDF, or BCRW PDF (Figure 7) using KL divergence and a ROC analysis. There was no central bias in the average image intensity map, and therefore, we did not pursue any further analysis with image intensity. Even when we removed images containing a horizon below the upper third of the image, we still could not find evidence of a central bias in image intensity maps (Supplementary Figure 3). In fact image intensity was a better predictor of the observed fixation locations for images with a horizon than images without a horizon.

Using KL divergence we found that there was no difference in the predictive ability of the central bias in observed fixations and the salience map (*ks*-test, *p* = 0.477), but the BCRW predicted fixations better than the central bias in fixation locations (*ks*-test, *p* < 0.001). Central biases in the BCRW and salience maps were significantly worse (*ks*-test, both *p'*s < 0.001) at predicting fixation location than the BCRW or salience, respectively.

A ROC analysis showed slightly different results. Central biases in the BCRW and salience maps could be used to discriminate between fixation locations and random locations better than chance (*z*-test, both *p'*s < 0.001), but the observed central bias in fixated locations was worse at discrimination than the BCRW or salience (*ks*-test, both *p's* < 0.001). The mean AUROC values using the average salience map, the average BCRW map, and the average observed fixation PDF biases as predictors of fixation locations were 0.640, 0.649, and 0.6591, respectively.

### Determining factors influencing the central bias in fixation locations

We hypothesized that a central bias in fixation locations is caused by the interaction between the arrangement of salient objects in complex scenes (i.e., photographer's bias) and the statistics of the eye movements. The BCRW is well-positioned to test this hypothesis because it allows for an isolation of factors influencing the central bias, such as a natural tendency to reorient the eyes toward the center of the stimulus, from the other statistics of eye movements. Further, data from the Shift Task can be used to determine whether the initial starting fixation position influences the central bias.

**Figure 8:**
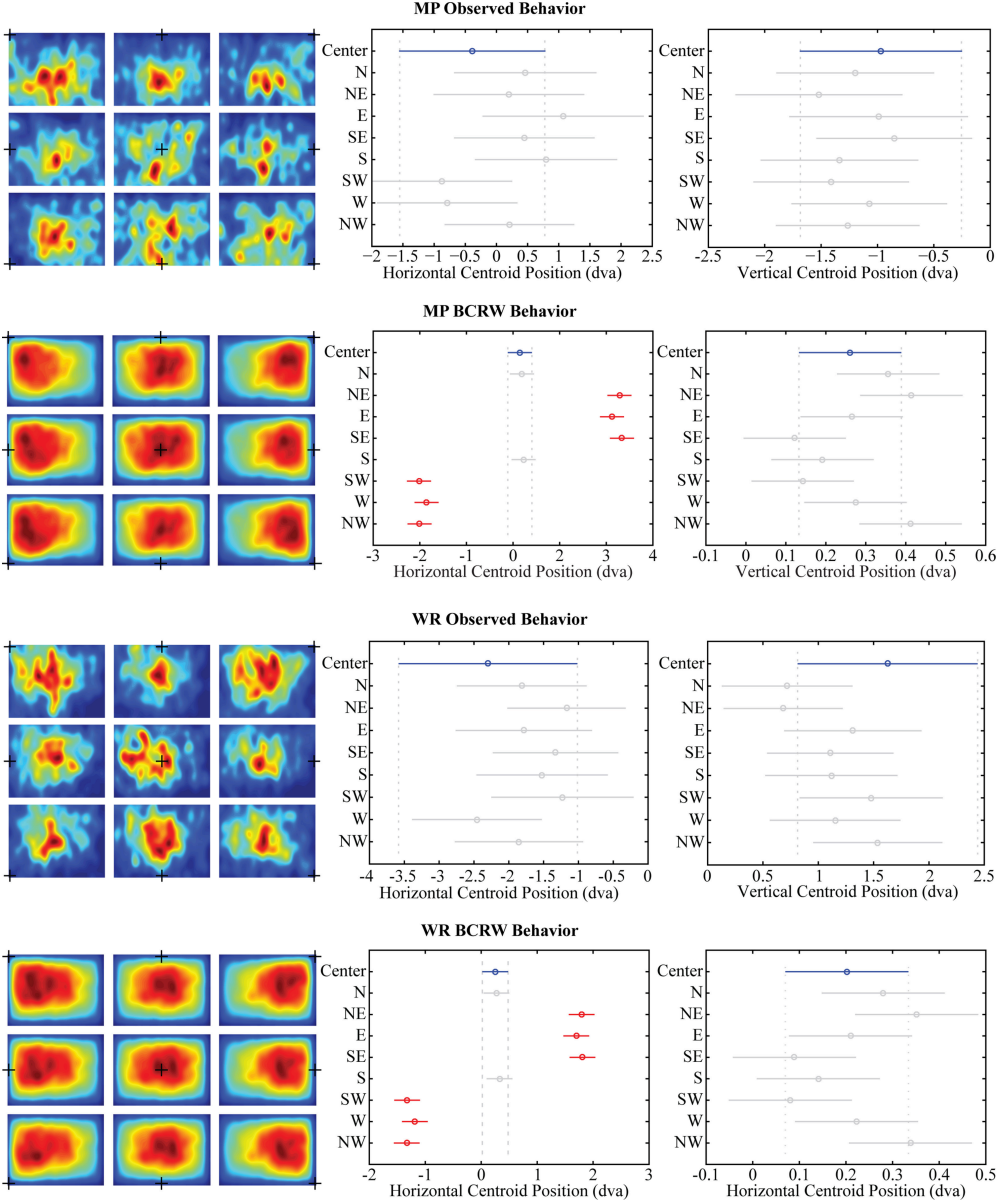
**Central bias**. Shifts in the position of the initial fixation cross (black + in all left panels) did not significantly displace the observed position of fixations (ANOVA, both *p's* > 0.11) for either monkey MP or monkey WR. However, when simulating viewing behavior with the BCRW, shifts in the position of the initial fixation cross resulted in significant displacements in the horizontal position of fixations (Middle panels, ANOVA corrected for multiple comparisons). Highlighted in blue is the centroid for the center fixation cross condition. Centroids significantly different than the centroid for the center fixation cross condition are highlighted in red. Significant displacements relative to the center fixation cross condition were not observed in the vertical shift conditions (Right panels). Error bars represent mean ± 95% confidence interval.

To measure the central bias, we calculated the centroid (center of mass) of the fixation PDF for each scan path on each image separately. In the observed data, we found there was no significant (one-way ANOVA, *p* > 0.11) displacement in the fixation centroid among the trials with different initial fixation cross positions (Figure 8). Further, changes in position of the image relative to screen center did not influence the central bias (one-way ANOVA, *p* > 0.10).

Interestingly, horizontal shifts in the initial starting position of the BCRW resulted in significant (one-way ANOVA, *p* < 0.05) displacements in horizontal position of the fixation centroid as compared to the central starting position condition. Vertical shifts in the initial starting position of the BCRW resulted in smaller, non-significant (ANOVA, *p* > 0.05) displacements in the vertical position of the fixation centroid as compared to the central starting position condition. Northern shifts in the initial starting position of the BCRW were significantly displaced north as compared to southern shifts in the initial starting position of the BCRW in fewer than half of all comparisons (one-way ANOVA corrected for multiple comparisons using Tukey-Kramer method, *p* < 0.05).

## Discussion

The use of natural stimuli along with natural viewing behavior is becoming widespread in neuroscience. Several recent studies provide evidence that complex scenes are useful for studying and diagnosing neurological disorders (Smith et al., 2006; Crutcher et al., 2009; Mannan et al., 2009; Tseng et al., 2013; Zola et al., 2013; Wang et al., 2015). Additional studies have elucidated novel neural responses in macaques freely viewing complex scenes (Killian et al., 2012; Hoffman et al., 2013; Jutras et al., 2013). Unfortunately, complex scenes are difficult to describe quantitatively and to parameterize. Further, complex stimuli are likely to elicit complex behavior that requires new analysis techniques. In order to fully interpret free viewing behavior, it is critical to understand what aspects of the stimuli guide this behavior.

We built the BCRW model to advance understanding of natural viewing behavior. The BCRW can aid in stimulus parameterization and can capture the complex behavior associated with viewing more complex stimuli. Here, we demonstrated that the BCRW can predict monkey viewing behavior for complex visual images better than chance. Additionally, the BCRW was able predict viewing behavior better than maps based on image salience or image intensity. Interestingly, the CRW, which did not contain a salience bias, was unable to predict viewing behavior as well as the BCRW. This suggests that *both* the statistics of eye movements and salience are important factors influencing fixation locations. We then demonstrated that the BCRW could be extended beyond the prediction of fixation locations. We showed that a central fixation bias cannot be explained by the interaction of the statistics of eye movements and the arrangement of salient objects in scenes.

The BCRW uses data derived from eye tracking in monkeys and combines these statistics with a salience map in a piece-wise fashion. The BCRW model incorporates statistics from individuals to account for inter- and intra-observer variability. A shortcoming of many deterministic attention models is the use of a winner-take-all algorithm to predict the next fixation, which assumes that the next fixation will always be in the same location regardless of the individual. However, there exists a great deal of individual variability in viewing behavior and each individual may view the same image differently across repeated presentations. The BCRW addresses this issue by directly predicting the probability of fixation at all locations. Regions with higher probabilities of a fixation will be fixated more often than regions with lower probabilities.

The mechanisms and circuits underlying attention and the control of eye movements are complex and not fully understood (Desimone and Duncan, 1995; Miller and Buschman, 2013). Instead of describing the mechanisms by which the brain executes attentional control, here, we built a more simplistic model of eye movements. The BCRW model is an appetitive foraging model in which the eyes are attracted to salience. Once the eyes fixate a location in the image, salience at that specific location becomes “consumed” until it recovers a specific time later at a rate dictated by the time constant of IOR. This consumption of salience may parallel extraction of visual information from fixated locations. The goal of the BCRW model is to parameterize viewing behavior during the viewing of complex scene stimuli at a phenomenological level. Future extensions of the BCRW could help us understand how certain mechanisms, such as IOR, are important for attention and how disease affects these mechanisms.

### The origin of the central bias in fixation locations

Whereas other models of viewing behavior often must incorporate additional measures to create a bias for fixations close to the center of an image (Parkhurst et al., 2002), the BCRW creates this bias without any additional influences. The average central biases in fixation locations were able to predict fixation locations better than chance. However, these average central biases were significantly worse at predicting fixation locations than salience or the BCRW model. Observation of individual scan paths supports the same conclusion. Individual scan paths did not strongly demonstrate a central bias but rather a strong bias toward salient regions. An apparent central bias in fixation locations was only revealed after averaging over a large number of scan paths.

Our last experiment aimed to understand which factors influence the central bias. The behavioral data showed that the position of the initial starting fixation and the position of the stimuli relative to the monkey and monitor are factors that do not strongly influence the central bias in fixation locations.

The BCRW was well-positioned to test the central bias hypothesis because parameters such as the initial starting position of the model could be easily manipulated. The BCRW could not reproduce the results found in the observed data suggesting that the central bias in fixation locations may not be caused solely by the interaction of oculomotor statistics with a central bias for salient regions of the image. Taken together with the behavioral results, these data suggest that the central bias is most strongly influenced by a natural tendency of the monkeys to re-orient their eyes toward the center of the stimulus.

Recent human studies exploring the nature of the central fixation bias have suggested that photographer's bias contributed prominently, and that the central bias was stronger during the beginning of a viewing period, with fixations distributed outside of the central area later in the viewing period (Tseng et al., 2009). In contrast, other studies have identified image and screen position as strongly influencing the central bias, particularly for early fixations (Bindemann, 2010). Further, Tatler suggested that observers demonstrate a central fixation bias because the center of the screen and stimulus offer a convenient reorienting location in that the eyes naturally relax to a forward position and observers are typically centered to the stimuli (Tatler, 2007). Our results are consistent with this later view and suggest that while the photographer's bias and eye movement statistics likely contribute to the central bias, monkeys have a general tendency to re-orient their eyes toward the center of the stimulus. Future experiments are necessary to fully understand the central bias of fixations. Additional modeling work offers a potential avenue for explaining some of these factors.

### Limitations of the BCRW model

Because the monkeys in our experiments viewed images for a cumulative looking time of 10 s, the apparent ability of the BCRW model and salience to predict fixations is less than some previous findings with shorter viewing periods (Judd et al., 2011; Wilming et al., 2011; Zhao and Koch, 2011). Here, we grouped viewing behavior statistics across the entire viewing period, but statistics including fixation duration and saccade amplitude change systematically over the viewing period. We also did not include higher-order relationships between preceding and subsequent fixation durations and saccade amplitudes (Tatler and Vincent, 2008). Incorporating these changes into the BCRW model may increase the predictive power of the BCRW. In addition, future investigations of the time-course of prediction by the BCRW could be useful in identifying periods of viewing behavior that are guided predominantly by bottom-up salience, as compared to other aspects of attentional control, including stimulus memory.

We used data from four monkeys to test the BCRW model and two monkeys to test the central bias hypothesis. While this sample size is small, it is of a size typical of many non-human primate studies. To alleviate statistical errors due to a small number of subjects we repeated the experiments over a large number of images and combined data across monkeys. Nonetheless, future studies with larger sample sizes will be useful in confirming the BCRW's interpretation of the salience maps.

Finally, a major limitation of the BCRW may derive from that fact that the model is an agent-based model in which the agent both diffuses randomly and is also heavily biased toward salient regions of the image. In short “saccades” in the BCRW do not always appear completely realistic and curve toward salient regions of the image. Ideally, the persistence term would maintain smooth saccade movements, but additional constraints may be necessary in order to create a more realistic model with increased predictive power. By averaging the results of the BCRW over 100 repetitions we may be removing the effects of this abnormality from the model.

## Conclusion

Bottom-up stimulus features such as salience predict free viewing behavior in monkeys. We can further increase the ability of bottom-up salience to predict behavior by interpreting the salience maps with a BCRW informed by viewing behavior statistics. We developed the BCRW to interpret salience maps, but the BCRW should be compatible with any algorithm used to calculate salience or other forms of attentional maps. Salience maps predict free viewing of complex scenes but may be insufficient for predicting viewing behavior in search-based tasks or viewing of familiar scenes where top-down mechanisms likely also influence viewing behavior. A potential solution to this limitation is the creation of hybrid models employing both bottom-up and top-down components of attention. Hybrid models could incorporate the BCRW as an eye movement model. Additional parameters, constraints, or layers could be added to the BCRW to increase the predictive power of the BCRW model.

The BCRW model can help in the creation and testing of novel behavioral tasks. The BCRW can be used to predict fixation locations, and in the case where there is a target or object of interest in a scene, the BCRW can predict the probability that a subject will look at the target or object of interest. Predictions like these can parameterize the non-intuitive aspects of behavioral tasks thus enabling the design of free-viewing tasks with consistent or incremental levels of difficulty.

## Author contributions

EB designed research; SK analyzed the data; and SK and EB wrote the paper.

### Conflict of interest statement

The authors declare that the research was conducted in the absence of any commercial or financial relationships that could be construed as a potential conflict of interest.

## Supplementary material

Supplementary Figure 1 **BCRW behavioral statistics**. The following viewing behavior statistics were incorporated into the BCRW: **(A)** fixation durations, **(B)** saccade durations, **(C)** the saccade angle leaving a fixation, **(D)** the [mean] eye movement velocity over time (dashed line is transition time from saccade to fixation), and **(E)** the relative weight of the previous movement (i.e., persistence). Blue lines represent behavioral statistics derived from the BCRW's scan paths and red lines represent behavioral statistics from monkey MP's scan paths. The probability distributions for fixation durations and saccade amplitudes were virtually identical. Due to rounding simulated scan paths to the nearest pixel in the BCRW, the BCRW's behavior diverged slightly from the observed behavior for saccade angles leaving a fixation and the velocity of eye movements over time.

Supplementary Figure 2 **Comparison of the scan paths simulated by the BCRW and CRW**. Top row: example scan paths from monkey IW, BCRW, and CRW for the same image. Fixation patterns appeared similar for the observed scan path and the BCRW. However, the CRW made fixations in random locations. Bottom row: as expected the average fixation PDF for the CRW appears relatively uniform except along the border of the image and did not reflect the observed fixation PDF well. BCRW scan paths continued to drift toward local salient peaks after fixation onset. The BCRW drifted slightly more than the CRW which did not contain a salience bias. Both the BCRW and CRW drifted more than the observed data. There was a small bump (~0.5 dva) in the observed data around 200 ms likely reflecting undetected microsaccades interrupting longer fixations.

Supplementary Figure 3 **Model fits for images with and without a horizon**. On average images that had a horizon (*n* = 84) had higher image intensity values toward the top of the image. No evident image intensity pattern was observed for images without a horizon (*n* = 204). The average salience map for images with a horizon had a bias toward the bottom of the screen while images that did not contain a horizon showed a strong central bias. The observed fixation PDF and BCRW map followed this downward shift in the salience map for images with a horizon. Image intensity was a better predictor of the observed fixation locations for images that had a horizon while the BCRW and salience maps predicted fixations for images with and without a horizon equally. Heat maps are scaled the same for images with and without a horizon. Error bars represent mean ± *SEM*.

## Abbreviations

AUROC: Area Under the Receiver Operating Characteristic Curve
BCRW: Biased Correlated Random Walk
CRW: Correlated Random Walk
dva: degrees of visual angle
KL divergence: Kullback-Leibler divergence
*ks*-test: Kolmogorov-Smirnov test
IOR: Inhibition of Return
PDF: Probability Distribution Function.
