# Supplementary figures and images for: Modeling Visual Exploration in Rhesus Macaques with Bottom-Up Salience and Oculomotor Statistics
Source: Front Integr Neurosci. 2016 Jun 30;10:23. doi: 10.3389/fnint.2016.00023 (PMC4928494; doi:10.3389/fnint.2016.00023)

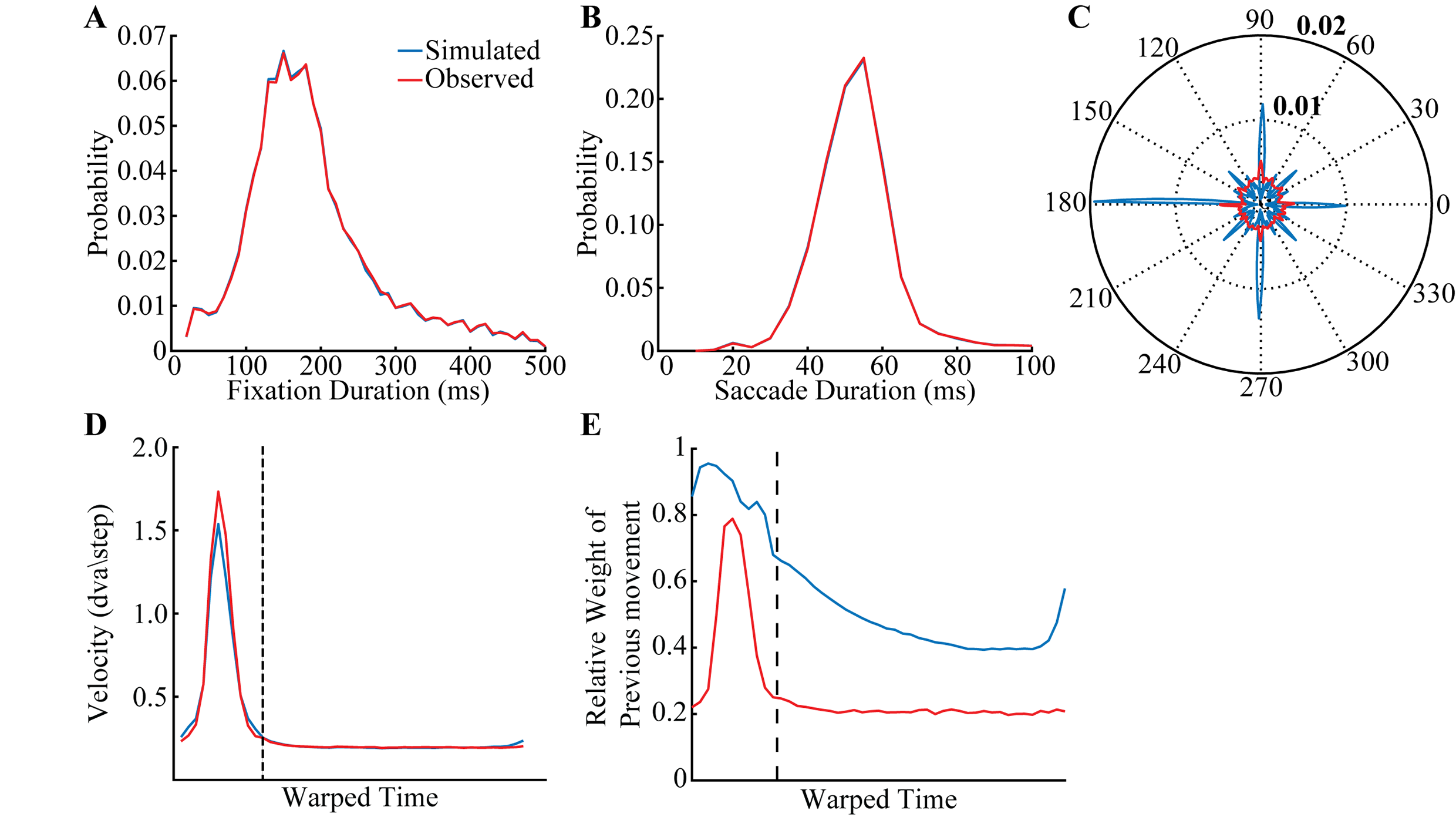

Supplement: Supplementary Figure 1 — BCRW behavioral statistics. The following viewing behavior statistics were incorporated into the BCRW: (A) fixation durations, (B) saccade durations, (C) the saccade angle leaving a fixation, (D) the [mean] eye movement velocity over time (dashed line is transition time from saccade to fixation), and (E) the relative weight of the previous movement (i.e., persistence). Blue lines represent behavioral statistics derived from the BCRW's scan paths and red lines represent behavioral statistics from monkey MP's scan paths. The probability distributions for fixation durations and saccade amplitudes were virtually identical. Due to rounding simulated scan paths to the nearest pixel in the BCRW, the BCRW's behavior diverged slightly from the observed behavior for saccade angles leaving a fixation and the velocity of eye movements over time. [file Image1.TIF]

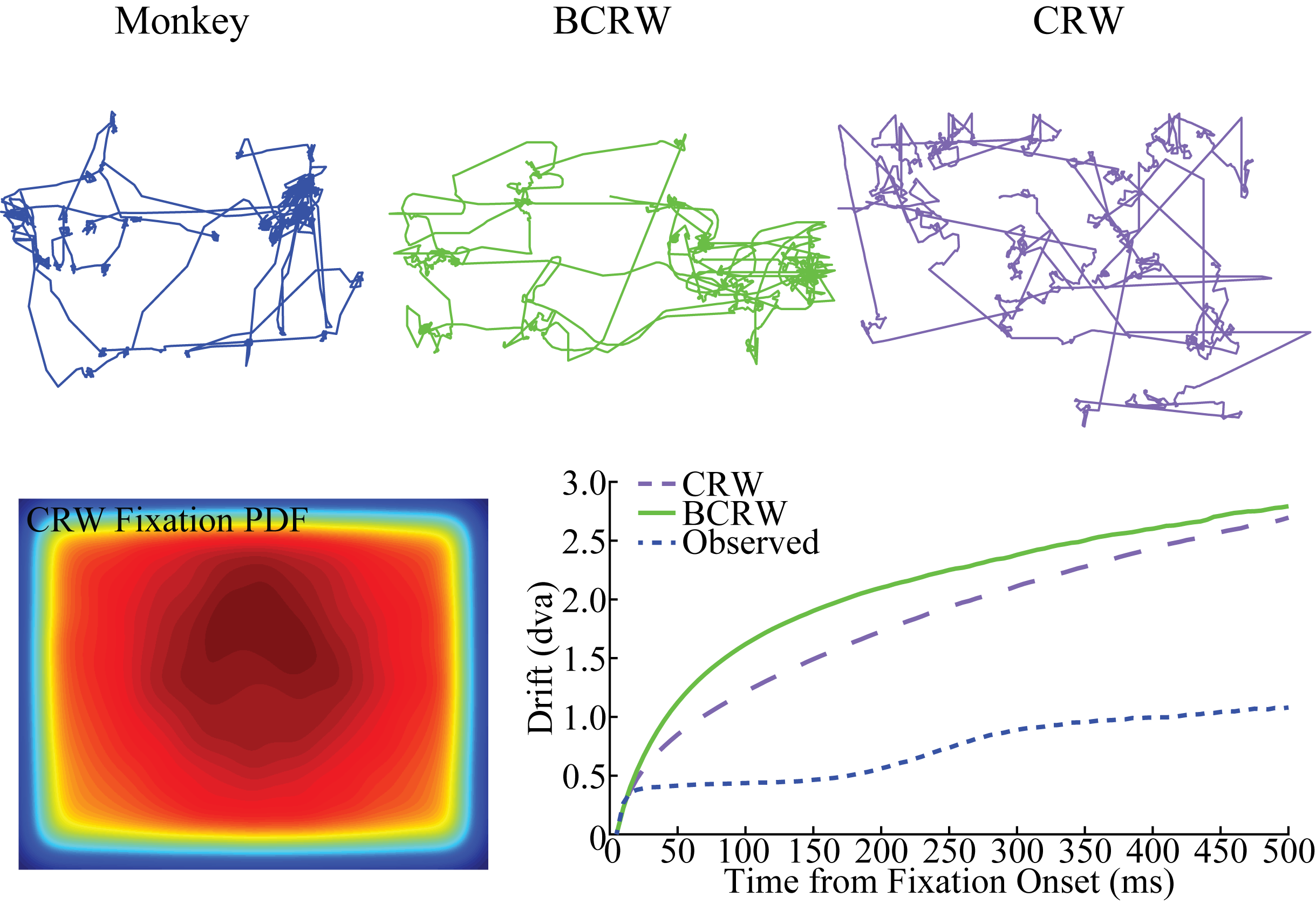

Supplement: Supplementary Figure 2 — Comparison of the scan paths simulated by the BCRW and CRW. Top row: example scan paths from monkey IW, BCRW, and CRW for the same image. Fixation patterns appeared similar for the observed scan path and the BCRW. However, the CRW made fixations in random locations. Bottom row: as expected the average fixation PDF for the CRW appears relatively uniform except along the border of the image and did not reflect the observed fixation PDF well. BCRW scan paths continued to drift toward local salient peaks after fixation onset. The BCRW drifted slightly more than the CRW which did not contain a salience bias. Both the BCRW and CRW drifted more than the observed data. There was a small bump (~0.5 dva) in the observed data around 200 ms likely reflecting undetected microsaccades interrupting longer fixations. [file Image2.TIF]

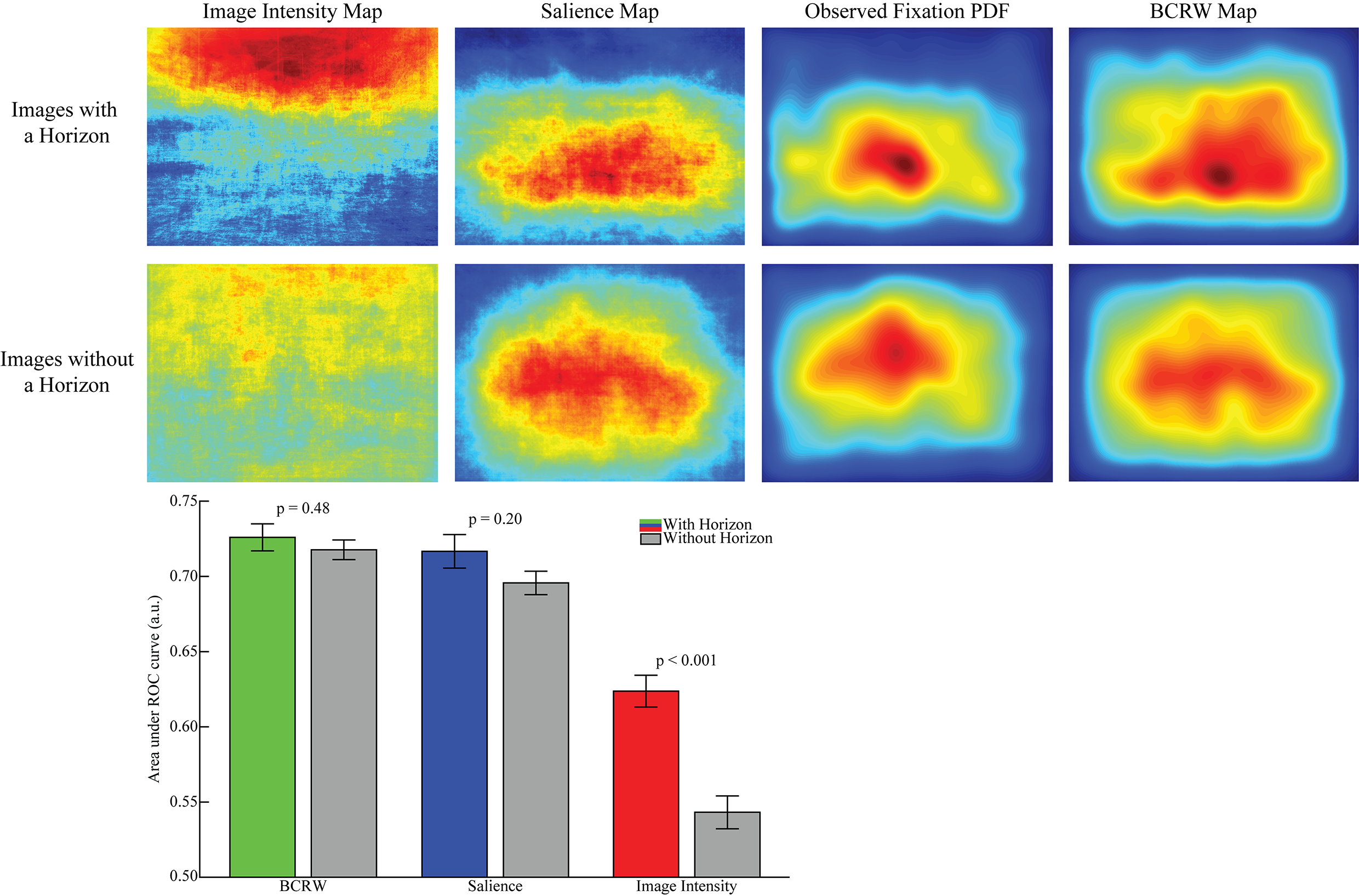

Supplement: Supplementary Figure 3 — Model fits for images with and without a horizon. On average images that had a horizon (n = 84) had higher image intensity values toward the top of the image. No evident image intensity pattern was observed for images without a horizon (n = 204). The average salience map for images with a horizon had a bias toward the bottom of the screen while images that did not contain a horizon showed a strong central bias. The observed fixation PDF and BCRW map followed this downward shift in the salience map for images with a horizon. Image intensity was a better predictor of the observed fixation locations for images that had a horizon while the BCRW and salience maps predicted fixations for images with and without a horizon equally. Heat maps are scaled the same for images with and without a horizon. Error bars represent mean ± SEM. [file Image3.TIF]
